# Single-cell study links metabolism with nutrient signaling and reveals sources of variability

**DOI:** 10.1186/s12918-017-0435-z

**Published:** 2017-06-05

**Authors:** Niek Welkenhuysen, Johannes Borgqvist, Mattias Backman, Loubna Bendrioua, Mattias Goksör, Caroline B Adiels, Marija Cvijovic, Stefan Hohmann

**Affiliations:** 1 Department of Chemistry and Molecular Biology, University of Gothenburg, SE-412 96 Gothenburg, Sweden; 2 Department of Mathematical Sciences, Chalmers University of Technology and the University of Gothenburg, SE-412 96 Gothenburg, Sweden; 3 Department of Physics, University of Gothenburg, SE-412 96 Gothenburg, Sweden; 4 Department of Biology and Biological Engineering, Chalmers University of Technology, SE-412 96 Gothenburg, Sweden

**Keywords:** Microfluidics systems, Glucose uptake, Non-linear mixed effect modelling, Dynamical modelling

## Abstract

**Background:**

The yeast AMPK/SNF1 pathway is best known for its role in glucose de/repression. When glucose becomes limited, the Snf1 kinase is activated and phosphorylates the transcriptional repressor Mig1, which is then exported from the nucleus. The exact mechanism how the Snf1-Mig1 pathway is regulated is not entirely elucidated.

**Results:**

Glucose uptake through the low affinity transporter Hxt1 results in nuclear accumulation of Mig1 in response to all glucose concentrations upshift, however with increasing glucose concentration the nuclear localization of Mig1 is more intense. Strains expressing Hxt7 display a constant response to all glucose concentration upshifts. We show that differences in amount of hexose transporter molecules in the cell could cause cell-to-cell variability in the Mig1-Snf1 system. We further apply mathematical modelling to our data, both general deterministic and a nonlinear mixed effect model. Our model suggests a presently unrecognized regulatory step of the Snf1-Mig1 pathway at the level of Mig1 dephosphorylation. Model predictions point to parameters involved in the transport of Mig1 in and out of the nucleus as a majorsource of cell to cell variability.

**Conclusions:**

With this modelling approach we have been able to suggest steps that contribute to the cell-to-cell variability. Our data indicate a close link between the glucose uptake rate, which determines the glycolytic rate, and the activity of the Snf1/Mig1 system. This study hence establishes a close relation between metabolism and signalling.

**Electronic supplementary material:**

The online version of this article (doi:10.1186/s12918-017-0435-z) contains supplementary material, which is available to authorized users.

## Background

Cells have developed an extensive network of signalling pathways in order to mediate appropriate responses to varying nutrient availability and concentrations in the surrounding environment. Glucose, a rapidly fermentable carbon source, is the preferred carbon source of *Saccharomyces cerevisiae* cells and regulates numerous nutrient signalling pathways [1, 2]. Glucose is taken up by the yeast cell through multiple hexose transporters, which have a broad range of different affinities and transport capacities. This enables the yeast cell to respond to a wide range of glucose concentrations [3, 4]. Glucose sensing pathways that employ membrane-localized receptors, such as in the Snf3-Rgt2 pathway, are relatively well understood. However, the sensing mechanism of intracellular glucose or metabolites of glycolysis is poorly explained [5, 6]. To study how metabolism is connected to these signalling pathways has proven to be a major challenge since it is difficult to uncouple signalling from metabolism.

The AMPK/SNF1 system controls energy homeostasis and is best known for its function in glucose signalling. The SNF1 protein kinase complex, which consists of three subunits, is activated by glucose depletion through phosphorylation [7, 8]. It is not well known how metabolism is connected with the activity of the SNF1 complex. When rapidly-fermentable sugars are available Snf1 becomes dephosphorylated. For the establishment of glucose repression only uptake and phosphorylation of glucose is required, but no further glucose metabolism [9]. Glucose repression is regulated, at least, at two different steps, i.e. control of Snf1 activation and its function on downstream targets such as Mig1 [10]. It has been suggested that the Snf1-Mig1 pathway works in a continuous on-off manner [11]. However, evidence has emerged that Mig1 shuttles in and out of the nucleus and shows transient behaviour at a single cell level [12, 13]. This indicates that the dynamics of the Snf1-Mig1 at single cell level is less simple than previously assumed.

To study the influence of glucose metabolism on the complex single cell dynamics of the Snf1 pathway we decided to control the uptake of glucose into the cell while employing a microfluidic system to control the extracellular glucose concentration. We show that glucose repression is regulated by glucose flux rather than the absolute glucose concentrations and that the Snf1-Mig1 system is closely regulated by glycolytic flux. In our experiments we observed cell-to-cell variability. To explain this variability, we developed a dynamical and nonlinear mixed effect model. Dynamical models of signalling pathways in yeast have previously been employed to describe the behaviour of populations of cells [14–19]. Nonlinear mixed effects (NLME) modelling is a theoretical approach that provides a framework to account for cell-to-cell variability [20]. NMLE modelling is traditionally used in pharmacokinetic and pharmacodynamics studies since it allows for the analysis of sparse and unbalanced datasets [21, 22]. NMLE has been proposed and used to model dynamic single cell data [20, 23]. Recently, a simple phenomenological model describing the Snf1-Mig1 pathway using NLME approach has been constructed, capturing dynamics of Mig1 localization, without taking into account parameter variabilities [24]. Our integrative approach reveals that the main source of variability is linked to transport of Mig1 in and out of the nucleus. Our experimental data indicate that rapid degradation and cell size cause no or little contribution to the cell-to-cell variability, while variation in expression and translation of the hexose transporters is a possible source of cell-to-cell variability.

## Results

### Single cell time-scale fluorescence microscopy enables dynamic studies of the Snf1-Mig1 pathway

A high control of the cell environment is needed in order to study nutrient responses on single cells. A microfluidics device allows for a fast and precise switch between different media and enables the nutrients composition in the media to be kept constant. Here we used a three inlet-channel microfluidics setup [25], to achieve a high control of the cell environment and to study the influence of glucose concentration on *Saccharomyces cerevisiae*. Upon deactivation of Snf1, Mig1 is dephosphorylated and subsequently moves into the nucleus and is therefore a suitable marker for real-time Snf1 activity [26, 27]. The nucleo-cytosolic shuttling of the transcription factor Mig1 fused to a Green Fluorescent Protein (GFP) served as single cell readout (Additional file 1: Figure S1).

**Fig. 1:**
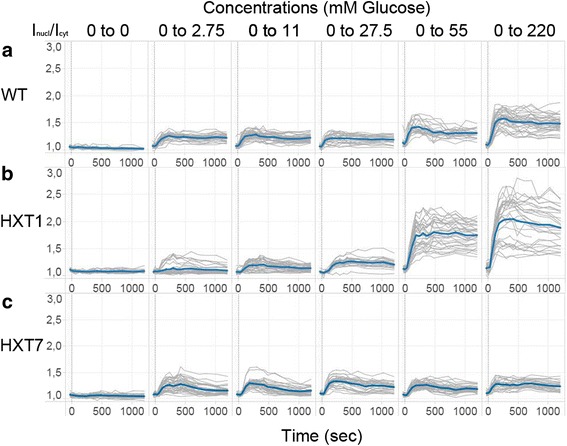
Localization of Mig1 over time expressed as nuclear fluorescence intensity divided by the cytosolic fluorescence intensity. Each graph depicts the results of one experiment. The y axis is distributed logarithmically. Each *grey line* represents the trace of one single cell and the average of all cells is represented with a thicker *blue line*. Between 22 to 41 cells were analysed in each experiment. The different strains are displayed vertically (wild type (WT) (**a**), HXT1 (**b**), HXT7 (**c**)) and the different concentrations are displayed horizontally

In the wild type (WT) Mig1 localized to the nucleus after being exposed to a glucose concentration of 2.75 mM (Fig. 1a and Additional file 2: Figure S2a). It has been shown that Mig1 is phosphorylated in this range of glucose concentration [28]. Following shifts to higher glucose concentrations more cells respond to the upshift and the Mig1 fluorescence intensity in the nucleus is stronger indicating that higher glucose concentrations result in a higher proportion of Mig1 molecules in the nucleus (Fig. 1a). The Snf1-Mig1 system responds to exposure to glucose and the degree of response is sensitive for the absolute glucose level which the cell is exposed to. These results are consistent with those of a similar study in *Saccharomyces cerevisiae* with a different genetic background [12]. The response to increased glucose concentrations occurs rapidly after upshift, pointing to the fast adaption of cells to nutrients in the environment.

### Glucose uptake through only low affinity transporters results in a strong response in the Snf1-Mig1 pathway

The data obtained from the wild-type strain raised the question whether glycolytic flux correlates directly with Snf1-Mig1 pathway activity. This would imply that the Snf1-Mig1 pathway is controlled by glucose metabolism by a quantitative sensor system. To address this question we chose to control the flux through glycolysis via the glucose uptake into the cell. A large set of isogenic strains expressing only a single hexose transporter is available [29]. We employed strains that express either a low affinity or high affinity glucose transporter, respectively, under the control of the promotor of a high affinity transporter.

Hxt1 is a low affinity transporter that is normally highly expressed under high glucose conditions; yeast cells expressing only *HXT1* displays a high glucose transport capacity and a higher *V*_*max*_ then the wild type strain [28, 29]. The HXT1 cells already display Mig1 nuclear accumulation when they were exposed to an upshift from 0 to 2.75 mM glucose as in the WT however the fraction of the whole population displaying nuclear localization never exceeds 50%. while for an upshift to 11 mM glucose a higher fraction of cells display nuclear accumulation but this nuclear accumulation never exceeds 80% (Additional file 3: Figure S3a). While in the WT for both the upshift to 2.75 mM and 11 mM more than 80% of the population displays nuclear accumulation. For the other upshift to higher glucose concentration nearly all cells display nuclear localization. The HXT1 strain displayed a large cell-to-cell variability of cells either responding or not responding to an upshifts of 2.75 mM, 11 mM, and 27.5 mM in glucose concentration in comparison with the WT cells (Fig. 1b). Only after an upshift to 27.5 mM glucose almost all the cells of the population show Mig1 localization in the nucleus. At higher glucose concentrations, 55 mM or 220 mM glucose, a higher proportion of Mig1 is localized in the nucleus than at lower concentrations. The upshifts to 55 mM and 220 mM result in a higher Mig1 nuclear accumulation compared to the WT and displays a higher cell variability compared to the other strains (Fig. 1b and Additional file 3: Figure S3b). The response times of Mig1 nuclear accumulation appear to be remarkably similar for all responding cells under all glucose concentrations, after 1 min the max is reached for the fraction of cells displaying Mig1 nuclear localization of all strains and all upshift conditions (Additional file 3: Figure S3a). Overall, the data shows that the response characteristics of the Snf1-Mig1 system correlates well with the kinetic characteristics of the Hxt1 transporter as the Snf1-Mig1 strain displays low response at an upshift to low glucose concentration but a strong response to upshift to high glucose concentrations.

### The high affinity transporter causes a weak Mig1 response to all glucose concentration upshifts

The Hxt7 high affinity transporter is highly expressed at very low glucose concentrations. The HXT7 strain displays a lower glucose uptake capacity than the HXT1 strain. Therefore the glucose uptake capacity is saturated at low glucose concentrations [28]. The majority of the population shows Mig1 nuclear localization after the cells are exposed to growth media containing glucose, in contrast with the low fraction of responders in the HXT1 strain (Fig. 1c and Additional file 2: Figure S2c). However, unlike in the WT and the HXT1 strain the response is very similar for all glucose concentrations, and the intensity of Mig1 in the nucleus is the same for all upshifts. Already at an upshift from 0 to 2.75 mM glucose Mig1 has reached a maximum Mig1 nuclear localization for the HXT7 strain. Hence, even in the HXT7 strain the Snf1-Mig1 response characteristic corresponds to the properties of the transport system with high affinity but low capacity.

### Neither regulated degradation nor cell size is a major contributor to cell heterogeneity

**Fig. 2:**
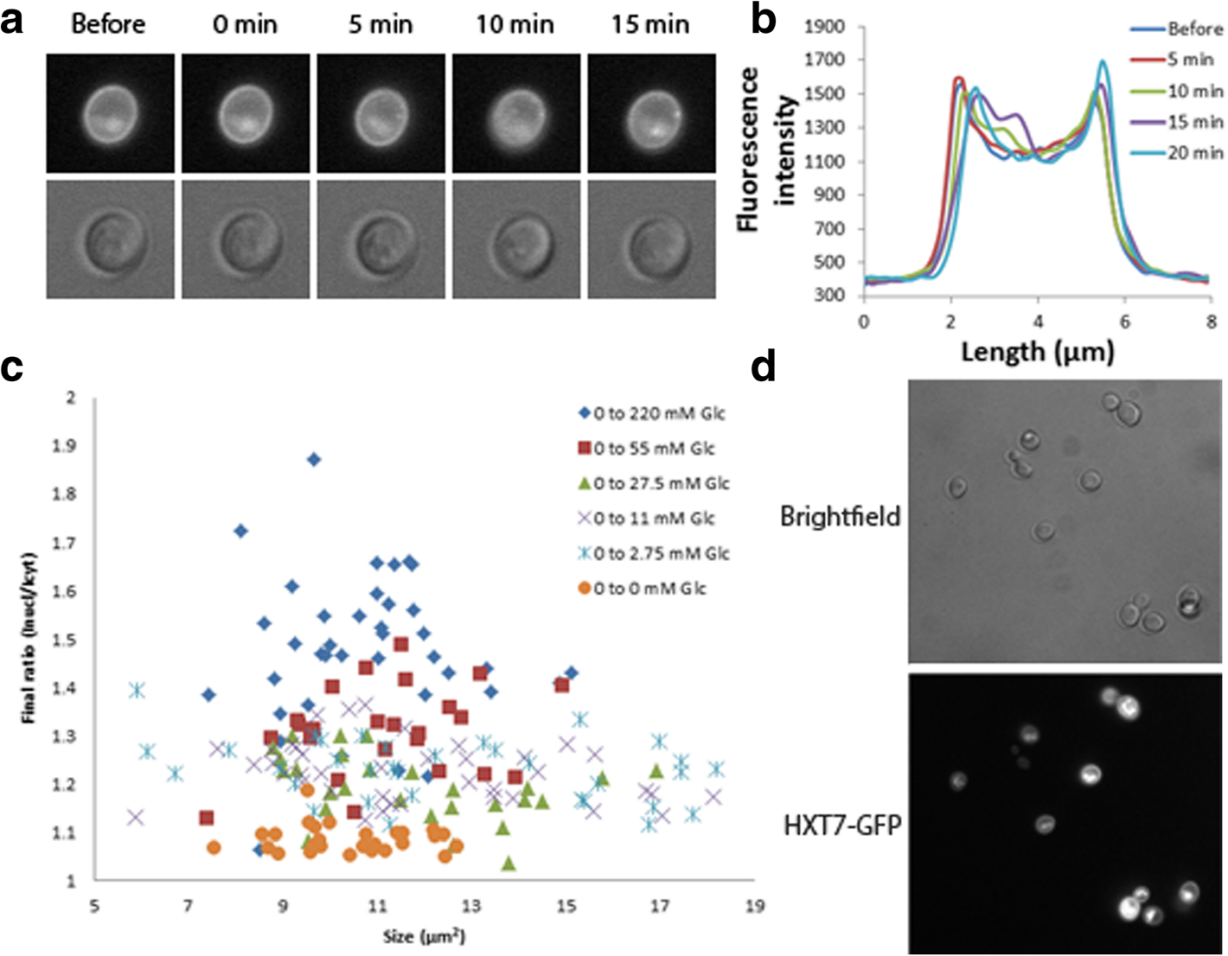
Study of the cell-to-cell variability observed in the Snf1/Mig1 system. (**a**)(**b**) Hxt7-GFP before and following a switch from ethanol media to media containing 220 mM glucose. (**a**) Time lapse microscopic images, *upper* images show HXT7-GFP, the *lower* images show brightfield. (**b**) Fluorescence intensity along an intersection through the yeast cells. The fluorescence intensity is higher at the points the *intersection line* crosses the cell membrane and does not change over time. The result of only one cell is displayed but multiple cells were analyzed and none of the cells showed a decrease in membrane localization of the Hxt7 transporter after 15 min following the shift to glucose media. (**c**) The ratio 15 min after glucose upshift plotted over the cell size for the HXT1 strain. The cell size is plotted on the x-axes. As a measurement for the Mig1-Snf1 pathway response we chose the Mig1 nuclear/cytosolic ratio. Upshifts to higher glucose concentration; 0 to 220 mM (*blue diamonds*), 0 to 55 mM (*red squares*) and 0 to 27.5 mM glucose (*green triangle*) result in higher final ratio while upshifts to lower glucose concentration 0 to 11 mM glucose (*purple crosses*), 0 to 2.75 mM (*blue stars*) and 0 to 0 mM glucose (*orange dots*) display a lower final ratio. (**d**) Hxt7-GFP pregrown overnight in 3% ethanol media. *Upper* image shows the bright field image, *lower* image shows the cellular distribution of Hxt7-GFP

Cell-to-cell variability in Mig1 localization upon changing glucose concentration has been reported [12, 13, 30]. These studies however do not examine the source(s) of the observed cell-to-cell variability, therefore we set out to explore the source(s) of the variation. It is known that the high affinity transporters Hxt7 and Hxt6 are internalized and degraded when cells are exposed to high concentration of glucose [31]. Degradation of Hxt7 requires inactivation of TORC1 [32, 33]. Also Hxt1 is actively internalized and degraded if glucose is depleted, an effect possibly mediated by downregulation of PKA [34]. Internalization of the hexose transporters for catabolic degradation could lead to a decrease of glucose import. We observed in the upshift experiments to higher glucose concentrations a slight decrease of the median after the nuclear localization reached its maximum value for all strains (Additional file 2: Figure S2). Therefore, we reasoned that rapid activity adjustments of the hexose transporters could impact the Mig1-Snf1 pathway. Since this drop in Mig1 nuclear localization differed between cells this mechanism could be a contributing factor to the observed cell-to-cell variability. We exposed yeast cells expressing Hxt7-GFP under the native promotor grown on 3% ethanol to 220 mM glucose and followed the localization of Hxt7-GFP for 15 min (Fig. 2a, Additional file 4: Figure S4). The data was quantified by measuring the fluorescence intensity along an intersection through the cell. We observed no significant change in the localization of Hxt7-GFP during the experiment (Fig. 2b).

We next asked whether cell size could influence the cell-to-cell variability. Fluctuations in cellular states, such as cell size, can cause extrinsic noise which could lead to the observed cell-to-cell variability [35]. We therefore decided to test the influence of cell size by plotting the response of the Snf1-Mig1 pathway over the cell size. As measurement for the Snf1-Mig1 pathway we used the Mig1 fluorescence intensity ratio 15 min after the upshift. The final ratio for the HXT1-strain did not show any correlation between the cell size and the Snf1-Mig1 pathway activity (Fig. 2c). Instead, the final ratio showed an even distribution around the average cell size with the values for the upshifts towards higher glucose concentration position higher along the y-axes. This result excludes cell size as a major determinant for the cell-to-cell variability on the relatively short time frame of the experiments.

### Stochastic expression is a plausible cause of cell-to-cell variability in the Mig1-Snf1 system

Cell-to-cell variability in dynamic adaptation responses might be caused by, among others, stochastic transcription activity [36]. Therefore, we reasoned that the expression pattern of the hexose transporters could lead to the cell-to-cell variability. We therefore grew a strain expressing Hxt7-GFP on 3% ethanol and followed the population distribution of Hxt7-GFP. The fluorescence intensity of Hxt7-GFP differed significantly between the cells (Fig. 2d). The lowest observed Hxt7-GFP fluorescence intensity was only 10% of the maximal observed fluorescence intensity of Hxt7-GFP. The amount of Hxt7 transporter molecules within each single cell varies and can therefore be a major contributor to the observed cell-to-cell variability. These results show that, under our experimental conditions it is likely that expression and translation of hexose transporters is a major contributor to the observed cell-to-cell variability.

### A mixed effect model suggests Mig1 dephosphorylation as a new regulatory step

**Fig. 3:**
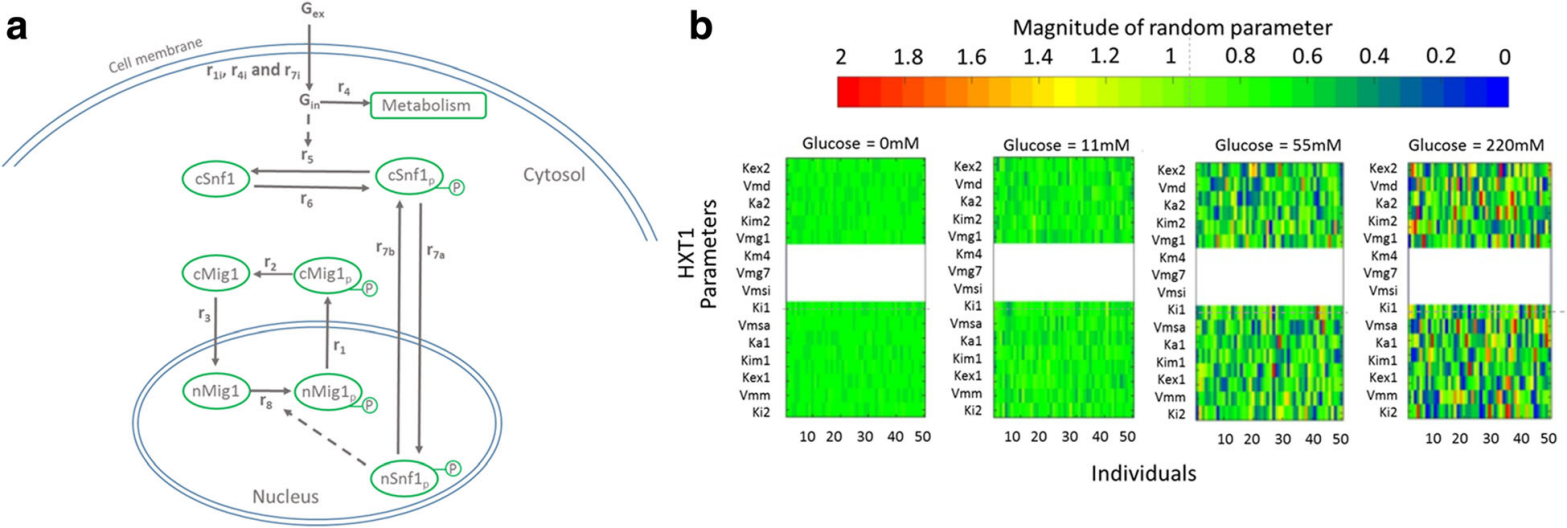
Dynamic and NLME modelling of the Snf1/Mig1 pathway. **a** Schematic representation of the model. The model consists of three main parts, namely the activity of glucose, the activity of Snf1 and the activity of Mig1. **b** Simulation of the distribution of the random parameters for the HXT1 strain. The columns indicate the extracellular glucose concentration, ranging from 0, 11, 55 to 220 mM which are illustrated from the *left* to the *right* in the figure. Each heat map is generated by drawing 50 mixed effect random terms, that is ***η*** ∼ **N**(0, ***σ***), corresponding to the parameter vectors from the generated parameter distributions for the various strains and glucose concentrations. The heat map displays various parameters on the y-axis, the individuals on the x-axis and the magnitude of the random terms are indicated by the colour scale shown above the figure. The colour scale ranges from 0 to 2 where a *red* colour corresponds to a high random term and a *blue* colour correspond to a low value of the random term. The white fields correspond to the parameters connected to the hexose transporters that are not active in HXT1 strains

To better understand the effect of glucose upshift on the Snf1-Mig1 pathway we developed a mathematical model of glucose flux which controls Snf1 phosphorylation and consequently Mig1 localization (Fig. 3a). The aim was to investigate if glucose uptake was able to regulate Mig1 localization by controlling only one step in the Snf1-Mig1 regulatory system. We assumed this step to be dephosphorylation of Snf1, since several publications identified this step to be controlled by the ADP/ATP ratio [37–40]. The ADP/ATP ratio is indirectly determined by glucose uptake and glycolysis, therefore the binding of ADP to the SNF1 complex could be the connection between glycolysis and the Snf1-Mig1 system. NLME modelling was implemented in order to simulate the dynamics of Mig1 localization for different yeast strains in various experimental conditions (Additional file 5: Figure S5). The model captures the characteristics of our experimental data (Fig. 1). By simulating parameters for multiple cells we could produce a distribution of the parameters and we compared this distribution between the Wild type, HXT1 and HXT7 strains (Additional file 6: Figure S6). The model predicts that the Vmsi, the parameter for Snf1 dephosphorylation, increases with upshifts to increasing glucose concentrations (Additional file 6: Figure S6a). However, there is no significant difference between the different strains. The model suggests Snf1 dephosphorylation to be active immediately after glucose is imported into the cell, but this process is influenced neither by glucose concentration nor by the strain it was simulated in. This suggests that Snf1 dephosphorylation is regulated more in an on/off fashion rather than in a dynamic fashion. However, the parameter for Mig1 dephosphorylation, Vmd, did display the characteristics of the different strain (Additional file 6: Figure S6b). At low glucose upshift the simulated Vmd parameters of the HXT7 strain were higher than the HXT1 parameters. Only at the higher upshift concentrations the Vmd parameters simulated for HXT1 strain where much higher than the Vmd parameters simulated for HXT7 strain. From this, the model suggested Mig1 dephosphorylation as a regulatory step which is controlled by glucose flux.

### A mixed effect model identifies hypothetical sources of variability in the Snf1-Mig1 regulatory module

Since our model takes cell-to-cell variability into account we could use it to identify which parameters display highest cell-to-cell variability and under which conditions. The wild type strain displayed increasing variance with upshift to the higher glucose concentrations 55 mM and 220 mM (Additional file 7: Figure S7). The highest variability was observed in the HXT1 strain with the upshift to the higher glucose concentrations (Fig. 3 and Additional file 7: Figure S7). The HXT1 transporter strain displayed a large cell-to-cell variability following shift to high glucose but a small cell-to-cell variability after upshift to low glucose. The simulated variance of the HXT7 strain was lower in comparison with the wild type and HXT1 strains and did not increase with upshift to higher glucose levels (Additional file 7: Figure S7). The simulated variance is in correlation with the observed variance seen in the experimental data (Additional file 3: Figure S3b). The model also allows us to predict the most important parameters that are the major contributor to the cell-to-cell variability. We compared the magnitude of perturbation of each parameter for the simulation of the wild type strain in the upshift from 0 to 220 mM glucose (Additional file 8: Table S1). A parameter which displays a higher perturbation error has a higher variability in that parameter. Parameters involved in dephosphorylation events have been ranked as low significance (Additional file 8: Table S1), suggesting that the dephosphorylation of Mig1 and Snf1 after glucose upshift is a minor contributor to the observed cell-to-cell variability. The parameters which display the highest variance are Kex2 and Kim2, which account for transport of Mig1 in and out of the nucleus. The respective perturbation errors for these two parameters are in the order of 10^−3^ while the other parameters have perturbation errors in the order of 10^−7^ and smaller. Indeed, it has been shown that movement of Mig1 in and out of the nucleus shows considerable variability between cells [12]. Those data suggest that variability in the nucleocytoplasmic transport of Mig1 would be the major contributor to cell-to-cell variability and not the dephosphorylation events following glucose upshifts.

## Discussion

It is well established that the Snf1-Mig1 system and hence the nuclear accumulation of Mig1 are controlled by the level of glucose in the growth medium. We have previously reported that glucose derepression senses glucose concentration in a highly dynamic fashion [12]. But it remained unclear whether the observed dynamics were correlated with adaptations of sugar transport and glycolytic metabolism. Previous modelling approaches have suggested the importance of the kinetics of the glycolytic flux in signalling of glucose [41]. To elucidate the influence of sugar transport on the Snf1-Mig1 pathway we studied the response of three different *S. cerevisiae* strains with different glucose uptake capacity when exposed to an upshift in glucose concentration. Our data show that yeast cells response rapidly to changes in glucose concentration and that there is little to no cell-to-cell variability in response time. Even between the strains and between glucose concentrations we observed no significant difference in response time. This indicates that yeast cells are programmed and determined to rapidly respond to a change in glucose concentration. This behaviour allows yeast cells to rapidly adapt to new environmental conditions and thereby to potentially outcompete other species. Although single yeast cells induced a response at almost the same time, there was difference between the cells in the magnitude of their response. Our data showed that the Mig1 localization pattern after glucose upshift correlates well with the glucose uptake characteristics of the respective yeast strains. It has already been shown that establishment of glucose repression is driven by sensing of an intracellular metabolite rather than extracellular glucose [42, 43]. Our single cell data confirms that glucose repression is sensed through an intracellular metabolite rather than extracellular glucose. It has been shown that in the strains we tested the reduced glucose uptake capacity results in reduced glycolytic rate [41]. The data indicate a closer link between the glucose uptake rate, which determines the glycolytic rate, and the activity of the Snf1/Mig1 system than previously anticipated. This indicates that the signal which originates from the glycolytic flux is very dynamic in response to the changing glycolytic rate. A source for this signal might be the ADP/ATP ratio [37, 38, 40]. Since, it has been reported that ADP binds to the regulatory subunit Snf4 and this binding leads to protection of the catalytic subunit Snf1 from dephosphorylation, which leads to increased Snf1 activity [38]. The turn-over of ADP to ATP or vice versa could be a sensor for the glycolytic rate. Also Hexokinase 2, an enzyme part of the glycolytic pathway, has been suggested to serve as a sensor for internal glucose by serving as a threshold for the interaction between Mig1 and Snf1 [44, 45].

Our data showed a considerable cell-to-cell variability in glucose sensing. This variability could have considerable impact on the Snf1/Mig1 pathway. We investigated the causes of the cell-to-cell variability that was observed in our initial experiments. Our results indicate that this behaviour is not caused by the size of the cell or rapid activity adjustments of the hexose transporters. A large variation is observed between the concentrations of Hxt7 in the cells and hence the high cell-to-cell variability can be caused by the expression and/or translation of hexose transporter Hxt7. In a yeast single cell study of the shift between sulphur sources it was observed that the transcriptional adaptation displayed a large cell-to-cell variability [36]. The variability in expression and translation of hexose transporters may cause a different uptake capacity within a population and consequently variability in further glucose metabolism. Such variability in glycolysis might lead to cells responding differently to nutritional changes and different subpopulations. Systems to restore unbalanced dynamics in glycolysis have already been reported [46].

Data obtained by single cell techniques coupled with mathematical modelling offer an opportunity to understand the variability within a population of cells. This work employs NLME Modelling in larger dynamical models providing a framework to deeper investigate and identify source of cell-to-cell variability in Snf1-Mig1 signalling pathway.

The variability in our model corresponded to that observed in our experimental data. Therefore, we can conclude that the implemented model can in fact account for the cell-to-cell variability of the nuclear/cytoplasmic Mig1 ratio. Our approach classifies certain parameters as low significant, having very small perturbation errors indicating that they could potentially be neglected without losing the predictive capability of the model (Additional file 8: Table S1). This partly explains that the simplified model proposed by Almqvist and colleagues [24] can capture the dynamics of the Mig1 localization, but fails to provide more information about the relationship between the parameters. To our knowledge, this is the first Mixed Effect Model which is complex enough to allow identification of sources of cell-to-cell variability in signalling pathways. Our model suggested a new regulatory step at the level of Mig1 dephosphorylation and this step would be controlled by glycolytic flux. It is known that Mig1 is dephosphorylated upon glucose addition leading to glucose derepression [28]. However, the glucose activated process which dephosphorylates Mig1 has not yet been clearly identified. For instance, it is unclear if Mig1 dephosphorylation is regulated by glucose or by a constitutive phosphatase counteracting Snf1 activity. Our computational approach suggests that Mig1 dephosphorylation is regulated and therefore probably an active step. The model suggest that dephosphorylation of Snf1 is regulated by absolute glucose concentrations while Mig1 dephosphorylation is regulated in a more dynamic way related to glucose flux. We were able to identify import and export of Mig1 in and out of the nucleus as a possible source for cell-to-cell variability. Both Mig1 import and export are regulated by glucose through phosphorylation and dephosphorylation of Mig1 [27]. Mig1 shuttles in and out of the nucleus regardless of the glucose concentration and FLIP/FRAP experiments have shown that there is a considerable cell-to-cell variability in Mig1 nuclear translocation [12]. Therefore, it is not entirely unexpected that this step is predicted to encompass a high cell-to-cell variability.

## Conclusions

This work links the glucose flux to Snf1-Mig1 signalling. Although the control of the Snf1-Mig1 regulatory module is complicated by crosstalk with other glucose sensing signalling systems, we suggest that glycolytic metabolic reactions are playing a major role in the regulation of Mig1 localization. We show that the initial response time of Snf1-Mig1 pathway displays no cell-to-cell variability. We further developed and presented a modelling approach which can model the cell variability observed in the data. Most importantly, we demonstrate the close correlation between glycolytic metabolism and glucose signalling metabolism.

## Methods

### Yeast strains

The strains employed were transformed with GFP-KanMx and mCherry hphNT1 using standard methods for yeast genetics and transformation: Yeast strains were grown to mid-log phase at 30 °C in YNB synthetic complete medium containing 1.7 g/l yeast nitrogen base, 5 g/l ammonium sulphate, 670 mg/l complete supplement mix and supplied with 3% ethanol. For the glucose upshift serial dilutions of a stock of 220 mM glucose YNB complete medium were made in order to ensure final glucose concentration ranging between 220 mM and 2.75 mM. A complete overview of the used strain can be found in Additional file 9: Table S2.

### Microfluidics

We employed Nrd1-RFP, a nuclear RNA-binding protein, as a nuclear marker. The ratio of Mig1-GFP within the nucleus to the Mig1-GFP throughout cells was used a quantifiable measure of Mig1 localization [47]. Strains were first pregrown in 3% ethanol, loaded on the microfluidic device and exposed to an upshift in glucose concentration, from 0 mM to 0 mM, 2.75 mM, 11 mM, 27.5 mM, 55 mM or 220 mM glucose which corresponds to respectively 0 to 0%, 0.05, 0.2, 0.5, 1 or 4%.To control the spatial and temporal changes of extracellular glucose concentration in the environment of the yeast cell we applied a three-channel microfluidic system that merge into one single channel [25]. We attached single cells to the surface of the single channel. By maintaining a constant flow of media through one of the three inlet channel we could expose the yeast cells to a certain concentration of glucose while acquiring time-lapse images. By analysing individual cells in these images with the Cellstat and CellStress software [48, 49], we could track the nuclear localization dynamics of Mig1 over time. For more details in on the microfluidics, imaging and data analysis see *Bendrioua* et al.*, 2014* [12] and Additional file 10.

### Data analysis

The boxplots, fraction and experimental coefficient of variation plots were generated in Matlab (MathWorks, MA).

### Model description

The presented model (Fig. 3a) consists of three modules: (1) activity of glucose, (2) the activity of Mig1 and (3) the activity of Snf1. The activity of Glucose includes the import of extracellular Glucose (Gex) into the cell and the degradation of intracellular Glucose (Gin) through the events of metabolism. The activity of Mig1 consists of the import and export of Mig1 into and out of the nucleus and the phosphorylation and dephosphorylation of Mig1. This activity is an irreversible cycle in which the Mig1 alternates between four different forms namely cytosolic Mig1 (cMig1), phosphorylated located in the cytosol Mig1 (cMig1p), Mig1 located in the nucleus (nMig1) and phosphorylated Mig1 located in the nucleus (nMig1p). The activity of Snf1 is divided into two subevents, firstly the phosphorylation and desphosphorylation of cytosolic Snf1 (cSnf1) resulting in phosphorylated cytosolic Snf1 (cSnf1p) and secondly the import and export of phosphorylated Snf1 located in the nucleus (nSnf1p). The set of Ordinary Differential Equations (ODEs) describing the dynamics of the system is listed below. Note that in Eq. (1), the three scalars HXT1a, HXT4a and HXT7a are introduced in order to account for the three data sets: HXT1, the HXT7 and the WT. They are represented as binary variables, where the value of 1 indicates their presence in the given reaction and 0 otherwise (for details see Additional file 10).

1 $$ \frac{{\mathrm{dG}}_{\mathrm{in}}}{\mathrm{dt}}=\left(\mathrm{HXT}1\mathrm{a}\cdot {\mathrm{r}}_{1\boldsymbol{i}}\right)+\left(\mathrm{HXT}4\mathrm{a}\cdot {\mathrm{r}}_{4\mathrm{i}}\right)+\left(\mathrm{HXT}7\mathrm{a}\cdot {\mathrm{r}}_{7\boldsymbol{i}}\right)-{\mathrm{r}}_4 $$

2 $$ \frac{\mathrm{dcSnf}1}{\mathrm{dt}}={\mathrm{r}}_5-{\mathrm{r}}_6 $$

3 $$ \frac{\mathrm{dcSnf}1\mathrm{p}}{\mathrm{dt}}={\mathrm{r}}_6-{\mathrm{r}}_5-\left({\mathrm{r}}_{7\mathrm{a}}-{\mathrm{r}}_{7\mathrm{b}}\right) $$

4 $$ \frac{\mathrm{dnSnf}1\mathrm{p}}{\mathrm{dt}}={\mathrm{r}}_{7\mathrm{a}}-{\mathrm{r}}_{7\mathrm{b}} $$

5 $$ \frac{\mathrm{dcMig}1\mathrm{p}}{\mathrm{dt}}={\mathrm{r}}_1-{\mathrm{r}}_2 $$

6 $$ \frac{\mathrm{dcMig}1}{\mathrm{dt}}={\mathrm{r}}_2-{\mathrm{r}}_3 $$

7 $$ \frac{\mathrm{dnMig}1}{\mathrm{dt}}={\mathrm{r}}_3-{\mathrm{r}}_8 $$

8 $$ \frac{\mathrm{dnMig}1\mathrm{p}}{\mathrm{dt}}={\mathrm{r}}_8-{\mathrm{r}}_1 $$

The collection of all the parameters, their connection to their respective reaction and the meaning of each reaction is listed in Additional file 11 Table S3.

### Non-linear mixed effect modelling

The dynamical model consists of 8 species, 12 reactions and 18 parameters. The vector consisting of these 18 parameters represents the overall dynamics of the entire population of cells and is denoted as fixed effect vector $\overset{-}{\theta}\in {\mathbb{R}}_{+}^{18}$. However, in order to account for the individuality we constructed a lognormal distribution from which each parameter vector $\theta \in {\mathbb{R}}_{+}^{18}$ representing the dynamics of an individual cell is drawn from. Thus by introducing a multivariate normally distributed variable, denoted as mixed *effect* vector *η* ∈ ℝ^18^ with zero mean and the corresponding covariance matrix *σ* ∈ *ℝ* ^18 × 18^ (*η* ∼ N(0, *σ*)), it is possible to construct the lognormal distribution which is summarized in Eq. 9:

9 $$ \theta =\overset{-}{\theta}\cdot \exp \left(\eta \right),\kern0.75em \eta \sim \mathrm{N}\kern0.15em \left(0,\sigma \right) $$

### Parameter estimation

For parameter estimation a continuous optimization method was implemented and has been conducted using the AMIGO toolbox [50] a software package within MATLAB that utilizes the built in function fmincon in combination with a multiple shooting technique [50]. For more information about the parameter estimation see Additional file 10.

### Parameter perturbation

Each parameter in the fixed effect parameter vector $\overset{-}{\theta}$ has been perturbed individually by multiplying the parameter of interest with a scalar of the value exp(*s* ^2^), denoted as ${\overset{-}{\theta}}_{per_i}$ where *i* = 1 ,  … ,18 is the index of the parameter that is being perturbed. Given the above notation, a measure of the change in the output in response to the perturbation in the model is given by

10 $$ {\mathrm{e}}_{\mathrm{i}}=\frac{\mid \mid \hat{\mathrm{y}}\left(\overset{-}{\theta}\right)-\hat{\mathrm{y}}\left({\overset{-}{\theta}}_{{\mathrm{per}}_{\mathrm{i}}}\right)\mid \mid }{\mathrm{l}},\kern2em \mathrm{i}\in \left\{1,\dots, 18\right\} $$

where *e*_*i*_ is the mean perturbation error of parameter *i* and *l* is the number of time points for which the output has been measured. Thus, a larger value of *e*_*i*_ would correspond to a greater significance of parameter *i* in the vector *θ* in terms of explaining the spread of the measured output.

## Additional files

Additional file 1: Figure S1. Sequential images of typical experiment. (PDF 365 kb)

Additional file 2: Figure S2. Boxplot overview of the upshift experiments on all the strains. (PDF 173 kb)

Additional file 3: Figure S3. Data analysis on upshift data. (PDF 133 kb)

Additional file 4: Figure S4. Study of the cell-to-cell variability observed in the Snf1/Mig1 system. (PDF 107 kb)

Additional file 5: Figure S5. The dynamics of the Mig1-quotient. (PDF 464 kb)

Additional file 6 **Figure S6.** Simulation of the spread in the two parameters Vmsi and Vmd. (PDF 128 kb)

Additional file 7: Figure S7. Simulation of the distribution. (PDF 452 kb)

Additional file 8: Table S1. The perturbation error. (PDF 53 kb)

Additional file 9: Table S2. *S. cerevisiae* strains used in this study. (PDF 26 kb)

Additional file 10: Supplementary methods on microfluidics device, microscopy, cell imaging, data analysis, model description, parameter estibation, and covariance matrix estimation and construction (PDF 220 kb)

Additional file 11: Table S3. A collection of all parameters in the model. (PDF 37 kb)

## Abbreviations

ADP: Adenosine Diphosphate
AMPK: AMP-activated protein kinase
ATP: Adenosine Triphosphate
GFP: Green fluorescent protein
NLME: Non-linear mixed effect
PKA: Protein kinase A
RFP: Red fluorescent protein
WT: Wild-type
