# Supplementary material for: Single-cell study links metabolism with nutrient signaling and reveals sources of variability
Source: BMC Syst Biol. 2017 Jun 5;11:59. doi: 10.1186/s12918-017-0435-z (PMC5460408; doi:10.1186/s12918-017-0435-z)
Supplement: Supplementary file 1 — Sequential images of typical experiment. (PDF 365 kb) [file 12918_2017_435_MOESM1_ESM.pdf]

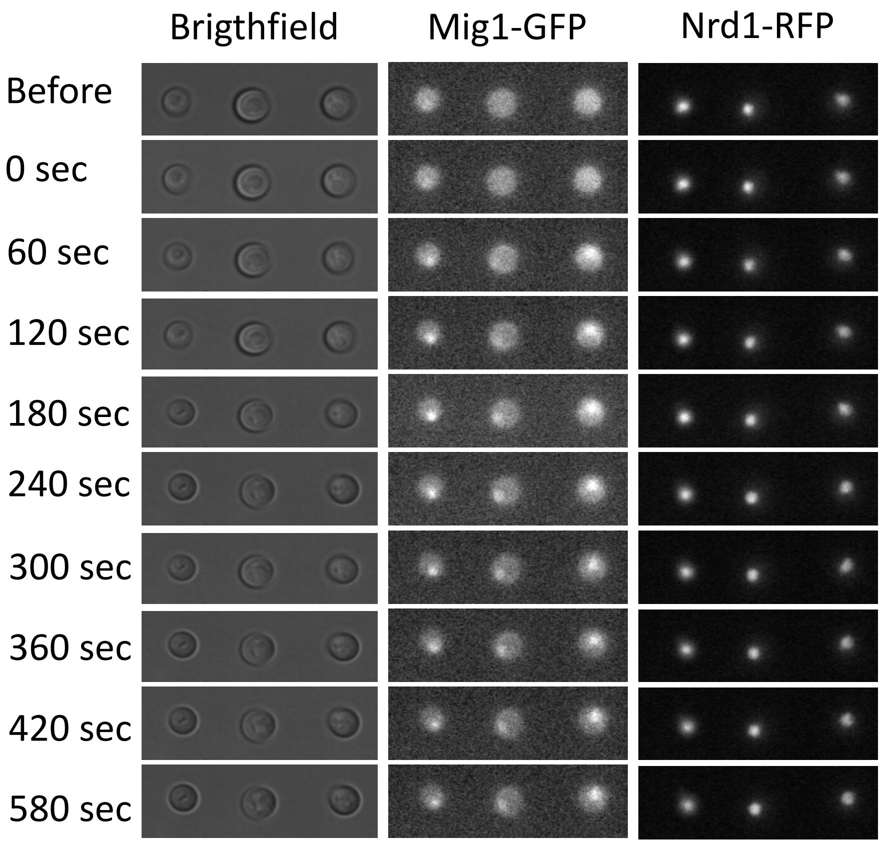

FigS1. Sequential images of typical experiment

Sequential images obtained during a microfluidic experiment of WT strain exposed to an upshift from 0 to 220 mM glucose. Brightfield images indicate were the cell membrane is situated, in the green field Mig1-GFP is observed and in the red field images (Nrd1-RFP) the nucleus is localized.
