# Supplementary material for: Single-cell study links metabolism with nutrient signaling and reveals sources of variability
Source: BMC Syst Biol. 2017 Jun 5;11:59. doi: 10.1186/s12918-017-0435-z (PMC5460408; doi:10.1186/s12918-017-0435-z)
Supplement: Supplementary file 2 — Boxplot overview of the upshift experiments on all the strains. (PDF 173 kb) [file 12918_2017_435_MOESM2_ESM.pdf]

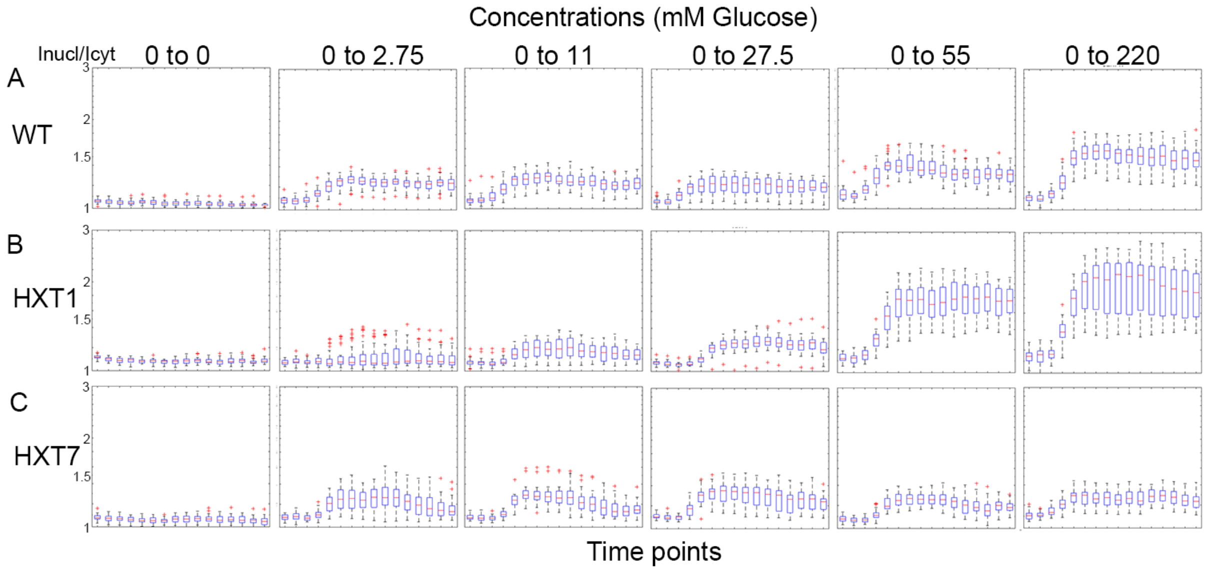

FigS2. Boxplot overview of the upshift experiments on all the strains.

These graphs show the spread by a boxplot of the single cells for every time point in the experiment. The bottom and the top of the box represent respectively the 25th and of 75th percentile and the middle band in the box represent the 50th percentile or median. The upper and the lower whisker represent a maximum of 1.5 IQR. The dots represent outliers. Each graph corresponds to one upshift experiments. The different strains are displayed vertically and the different concentrations are displayed horizontally. The wild-type strain displays the lowest spread of the measurements.
