# Supplementary material for: Single-cell study links metabolism with nutrient signaling and reveals sources of variability
Source: BMC Syst Biol. 2017 Jun 5;11:59. doi: 10.1186/s12918-017-0435-z (PMC5460408; doi:10.1186/s12918-017-0435-z)
Supplement: Supplementary file 3 — Data analysis on upshift data. (PDF 133 kb) [file 12918_2017_435_MOESM3_ESM.pdf]

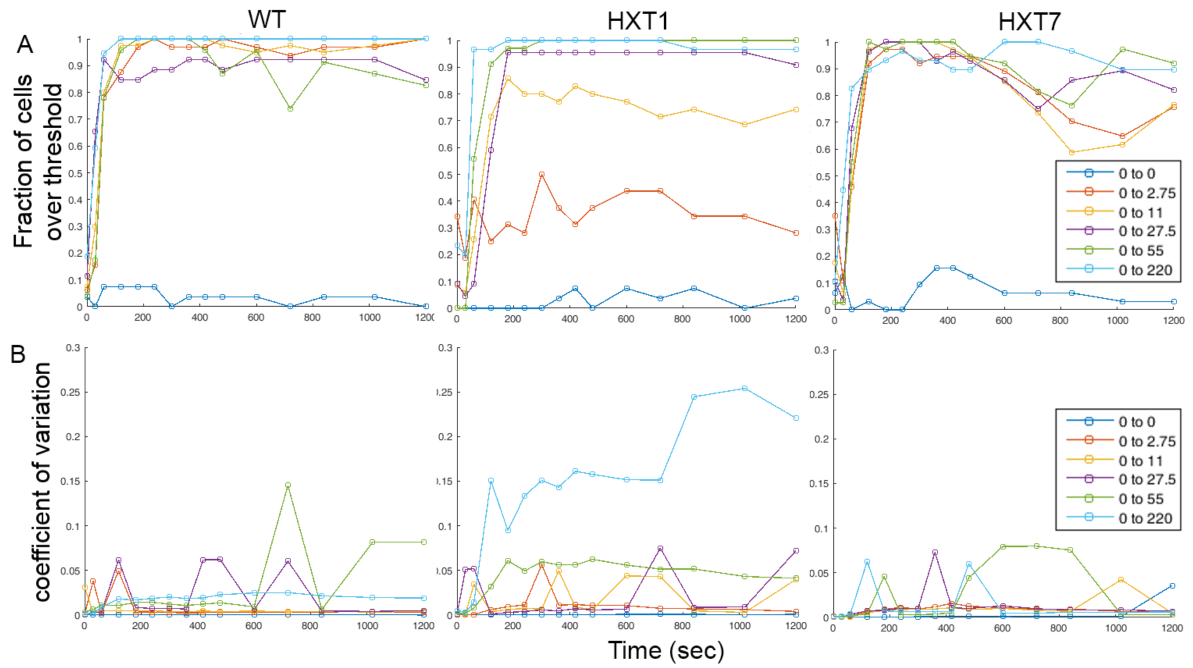

FigS3. Data analysis on upshift data.

Fraction of cells displaying nuclear localization over time. Nuclear localization is defined by cells which nuclear fluorescence intensity exceeds the mean nuclear intensity plus standard deviation of the mean nuclear intensity at timepoint zero. (Panel A). The coefficient of variation per experiment over time per experiment (Panel B)
