# Supplementary material for: Single-cell study links metabolism with nutrient signaling and reveals sources of variability
Source: BMC Syst Biol. 2017 Jun 5;11:59. doi: 10.1186/s12918-017-0435-z (PMC5460408; doi:10.1186/s12918-017-0435-z)
Supplement: Supplementary file 4 — Study of the cell-to-cell variability observed in the Snf1/Mig1 system. (PDF 107 kb) [file 12918_2017_435_MOESM4_ESM.pdf]

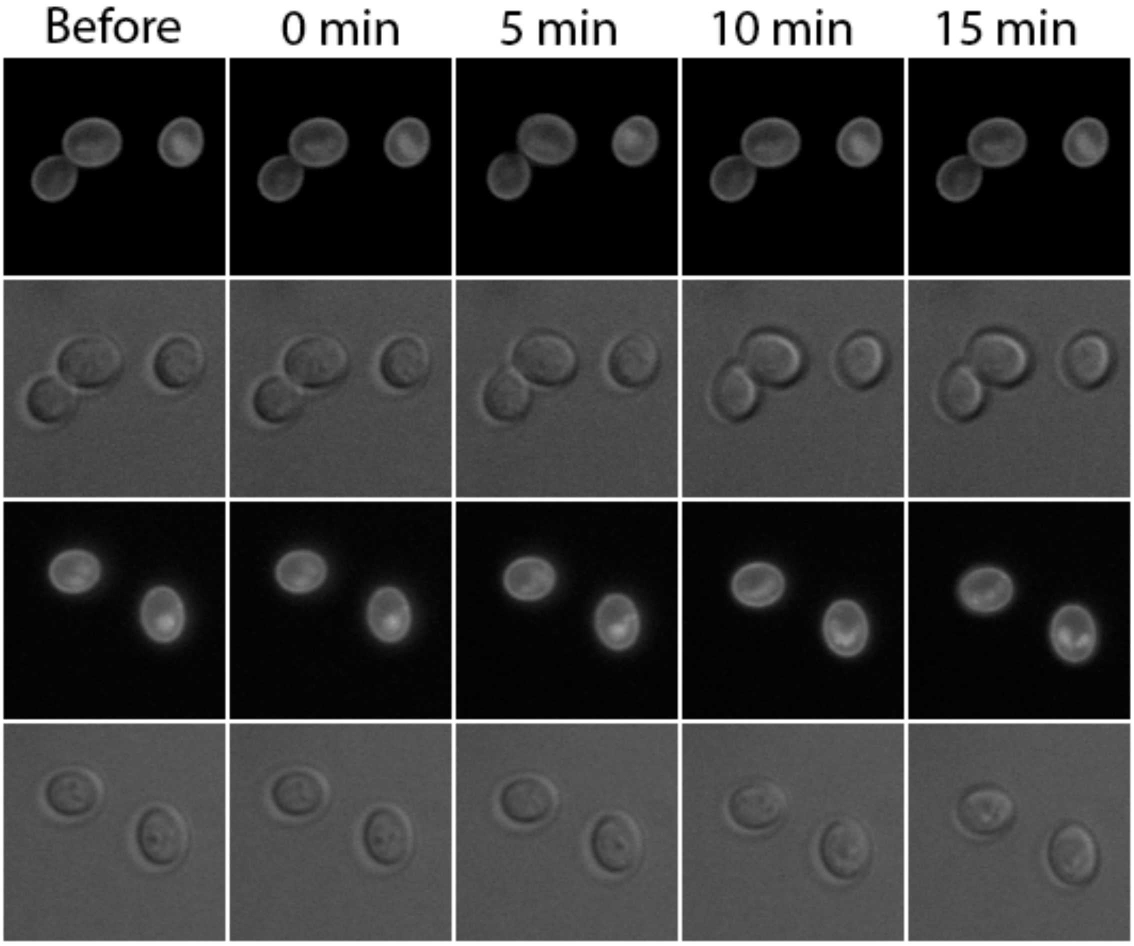

FigS4. Study of the cell-to-cell variability observed in the Snf1/Mig1 system.

Hxt7-GFP before and following a switch from ethanol media to media containing 220mM glucose. Time lapse microscopic images, upper images show HXT7-GFP, the lower images show brightfield**.**
