# Supplementary material for: Single-cell study links metabolism with nutrient signaling and reveals sources of variability
Source: BMC Syst Biol. 2017 Jun 5;11:59. doi: 10.1186/s12918-017-0435-z (PMC5460408; doi:10.1186/s12918-017-0435-z)
Supplement: Supplementary file 5 — The dynamics of the Mig1-quotient. (PDF 464 kb) [file 12918_2017_435_MOESM5_ESM.pdf]

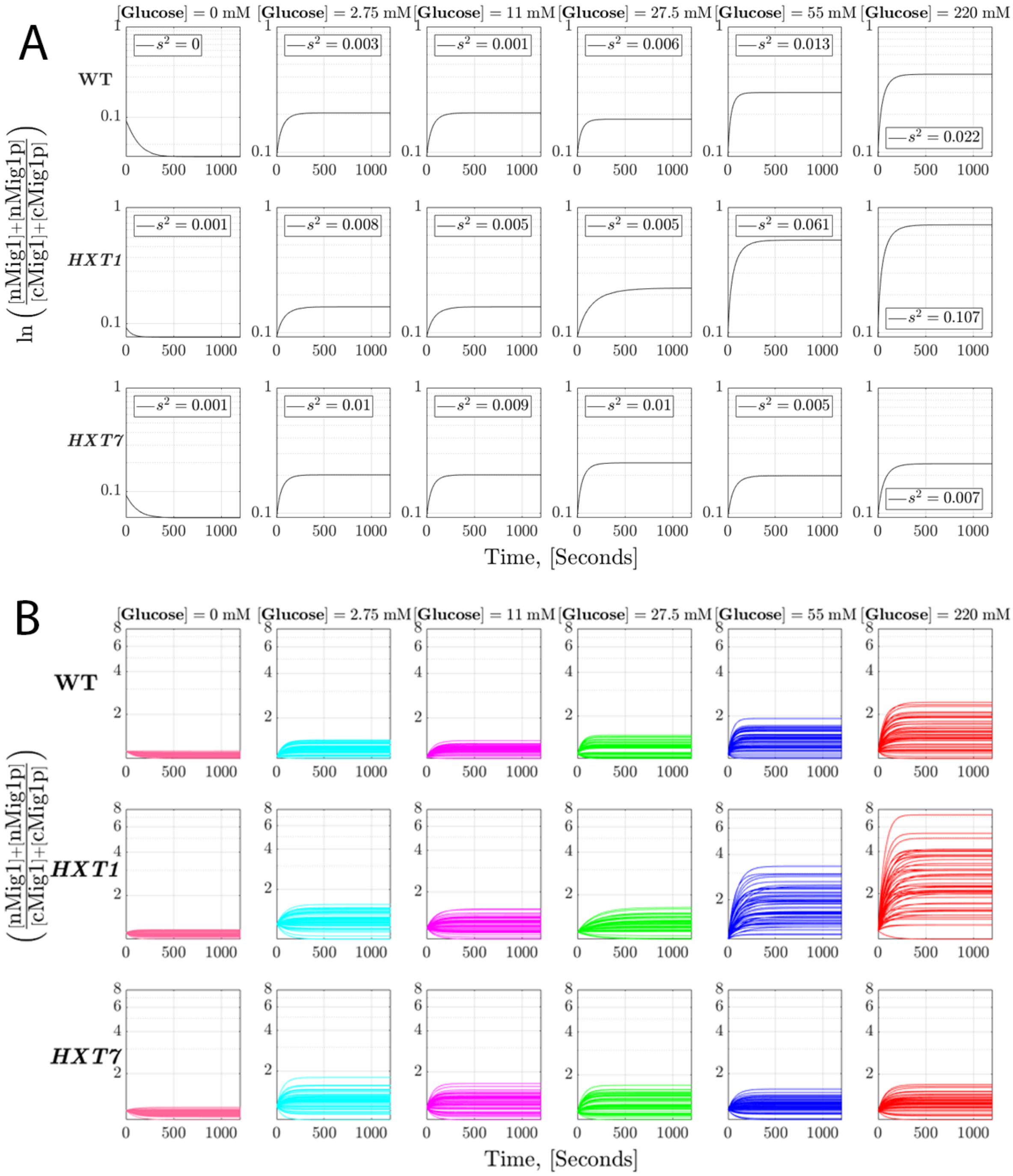

FigS5. The dynamics of the Mig1-quotient

The dynamics of the Mig1-quotientwith the estimated fixed effect parameter θ (Panel A), mixed effect parameter θ and the estimated covariance matrix σ for the various test conditions (Panel B). In all subfigures, the logarithmic y-axis represents the simulated output, the Mig1 quotient, while the time (in seconds) is displayed at the x-axis. The rows indicate the yeast strain while the column indicates the external glucose concentration. The three yeast strains are (from top to bottom) denoted WT, HXT1 and HXT7, while the six different external glucose concentrations (from left to right) are 0, 2.75, 11, 27.5, 55 and 220 mM.
