# Supplementary material for: Single-cell study links metabolism with nutrient signaling and reveals sources of variability
Source: BMC Syst Biol. 2017 Jun 5;11:59. doi: 10.1186/s12918-017-0435-z (PMC5460408; doi:10.1186/s12918-017-0435-z)
Supplement: Supplementary file 6 — Figure S6. Simulation of the spread in the two parameters Vmsi and Vmd. (PDF 128 kb) [file 12918_2017_435_MOESM6_ESM.pdf]

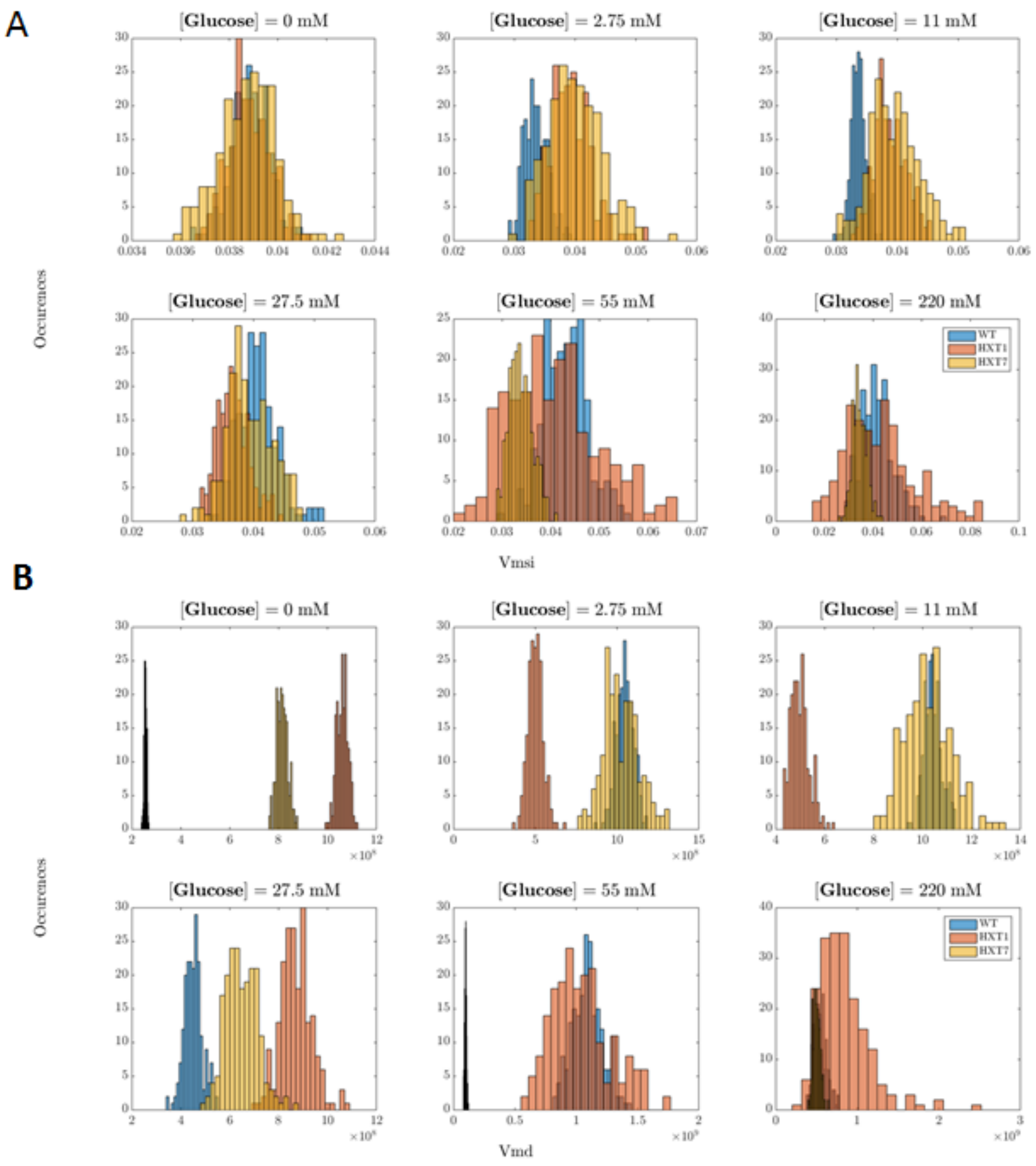

FigS6. Simulation of the spread in the two parameters Vmsi and Vmd.

(A) illustrates six histograms for the parameter Vmsi; (B) illustrates six histograms for the parameter Vmd. Each histogram corresponds to the spread of the given parameter for a specific external glucose concentration. On the y-axis of the histogram the number of occurrences is plotted against the actual value of the parameter on the x-axis. The histograms are generated by drawing 50 individuals in each strain and then gathering values of similar range into one bin where the height of the bin indicates the number of individual parameters within the range in question and the width of the bin indicates the range of the drawn parameter values. In all subfigures in the illustrated histograms, the parameters estimated for the WT data is illustrated in blue, the parameters estimated for the HXT1 data is illustrated in red and the parameters estimated for the HXT7 data is illustrated in yellow.
