# Supplementary material for: Single-cell study links metabolism with nutrient signaling and reveals sources of variability
Source: BMC Syst Biol. 2017 Jun 5;11:59. doi: 10.1186/s12918-017-0435-z (PMC5460408; doi:10.1186/s12918-017-0435-z)
Supplement: Supplementary file 7 — Simulation of the distribution. (PDF 452 kb) [file 12918_2017_435_MOESM7_ESM.pdf]

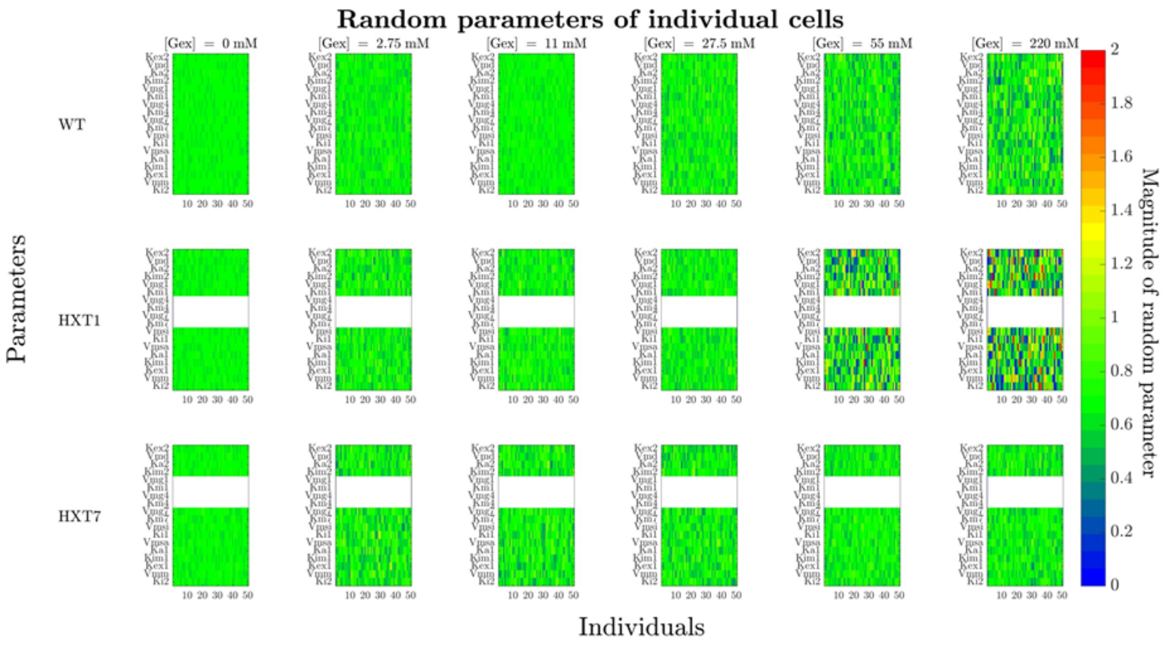

FigS7. Simulation of the distribution

Simulation of the distribution of the random parameters for the individual cells. The figure is divided into 18 subfigures structured in three rows and six columns. The rows indicate the various strains, namely WT, HXT1 and HXT7 respectively. The columns indicate the extracellular glucose concentration, ranging from 0, 2.75, 11, 27.5, 55 to 220 mM which are illustrated from the left to the right in the figure. Each heat map is generated by drawing 50 mixed effect random terms, that is $\eta \sim N(0, \sigma)$, corresponding to the parameter vectors from the generated parameter distributions for the various strains and glucose concentrations. The heatmap has the various parameters on the y-axis, the individuals on the x-axis and the magnitude of the random terms are indicated by the colour scale shown on the right in the figure. The colour scale ranges from 0 to 2 where a red colour corresponds to a high random term and a blue colour correspond to a low value of the random term. In the HXT1 and HXT7 strain, the white fields correspond to the parameters connected to the hexose transporters that are not active in these strains. As can be seen, for high glucose concentrations the spread (depicted in the range of the colours in the plot) is highest in the HXT1 strain, second highest in the WT strain and lowest in the HXT7 strain in the case of high extracellular glucose concentrations.
