# Supplementary material for: Single-cell study links metabolism with nutrient signaling and reveals sources of variability
Source: BMC Syst Biol. 2017 Jun 5;11:59. doi: 10.1186/s12918-017-0435-z (PMC5460408; doi:10.1186/s12918-017-0435-z)
Supplement: Supplementary file 8 — The perturbation error. (PDF 53 kb) [file 12918_2017_435_MOESM8_ESM.pdf]

Parameter	Perturbation Error, e	Level of significance
Kim2	$3.51 \cdot 10^{-3}$	High
Kex2	$3.182 \cdot 10^{-3}$	High
Vmsa	$2.6355 \cdot 10^{-7}$	Middle
Kim1	$2.5794 \cdot 10^{-7}$	Middle
Vmg4	$2.5509 \cdot 10^{-7}$	Middle
Vmg7	$2.4821 \cdot 10^{-7}$	Middle
Vmm	$2.4738 \cdot 10^{-7}$	Middle
Vmg1	$2.4735 \cdot 10^{-7}$	Middle
Kex1	$2.4545 \cdot 10^{-7}$	Middle
Km7	$2.4539 \cdot 10^{-7}$	Middle
Ka1	$2.4465 \cdot 10^{-7}$	Middle
Ki2	$2.1965 \cdot 10^{-7}$	Middle
Km1	$5.6637 \cdot 10^{-10}$	Low
Km4	$7.4751 \cdot 10^{-12}$	Low
Vmsi	$2.484 \cdot 10^{-13}$	Low
Vmd	$9.5691 \cdot 10^{-14}$	Low
Ki1	$4.9288 \cdot 10^{-14}$	Low
Ka2	$1.0047 \cdot 10^{-15}$	Low

Table S1. The perturbation error

The perturbation error, depicted in the middle column, in response to a perturbation in each parameter, depicted in the left column. Furthermore, the right column contains a classification in terms of the significance of the magnitude of the perturbation error where the three classification levels are high, middle and low. The parameters that have been perturbed are the parameters that were estimated in the wild type data in 220 mM extracellular glucose. Each parameter has been perturbed by a factor $exp\ (s^2)$ where $s^2 = 0.022$.
