# Supplementary material for: Single-cell study links metabolism with nutrient signaling and reveals sources of variability
Source: BMC Syst Biol. 2017 Jun 5;11:59. doi: 10.1186/s12918-017-0435-z (PMC5460408; doi:10.1186/s12918-017-0435-z)
Supplement: Supplementary file 9 — S. cerevisiae strains used in this study. (PDF 26 kb) [file 12918_2017_435_MOESM9_ESM.pdf]

Strain name	genotype	construct	source
CEN.PK2-1C83	MATa MAL2-8c<br><br>SUC2	*Mig1-GFP-KanMx Nrd1-mCherry-hphNT1*	K.D. Entian, Frankfurt am Main, Germany, Fluorescence tag: this study
KOY.HXT1P	KOY.VW100 integration into cassette: *HXT7 prom-HXT1-HXT7 term ura3-52::URA3*	*Mig1-GFP-KanMx Nrd1-mCherry-hphNT1*	[1], fluorescence tag: this study
KOY.HXT7P	KOY.VW100 integration into cassette: *HXT7 prom-HXT7-HXT7 term ura3-52::URA3*	*Mig1-GFP-KanMx Nrd1-mCherry-hphNT1*	[1], fluorescence tag: this study
KOY.TM6*P	KOY.VW100 integration into cassette: *HXT7 prom-TM6*-HXT7 term ura3-52::URA3*	*Mig1-GFP-KanMx Nrd1-mCherry-hphNT1*	[1], fluorescence tag: this study
HXT7-GFP	MATa MAL2-8c<br><br>SUC2 HXT7-GFP::Hix3MX6	*Mig1-GFP-KanMx Nrd1-mCherry-hphNT1*	[2], transfer of construct to new background: this study

Table S2. *S. cerevisiae* strains used in this study
