# Supplementary material for: Single-cell study links metabolism with nutrient signaling and reveals sources of variability
Source: BMC Syst Biol. 2017 Jun 5;11:59. doi: 10.1186/s12918-017-0435-z (PMC5460408; doi:10.1186/s12918-017-0435-z)
Supplement: Supplementary file 10 — Supplementary methods on microfluidics device, microscopy, cell imaging, data analysis, model description, parameter estibation, and covariance matrix estimation and construction (PDF 220 kb) [file 12918_2017_435_MOESM10_ESM.pdf]

Supplementary methods

Microfluidics system

The cell suspension was introduced into the lower inlet channel, the middle channel contained the growth medium and the top inlet channel contained the media with the new glucose conditions. Individual yeast cells were captured with optical tweezers and positioned in a 6x5 cell array. The device was pretreated with concanavalin A resuspended in 10 mM Tris-HCL and 100 mM NaCl (pH 8.0) for at least 30 min to make the cell stick to the device surface. The three inlets were connected by polytetrafluoroethylene tubing (Cole-Parmer, Vernon Hills, IL), which were attached to 250 µl Hamilton glass syringes (Hamilton Co., Switzerland) through needles. The flow was controlled with syringe pumps (CMA Microdialysis AB, Sweden). Both the lower and the middle inlet were connected to the same pump (pump 1), whereas the upper inlet was connected to a second pump (pump 2). At the start of the experiment, pump 1 was set to 80 nl/min, and pump 2 was set to 40 nl/min. To perform the medium shift pump 1 was turned off and pump 2 was switched to 1000 nl/min. The flow changes and image acquisition events were synchronized by OpenLab (Improvision Inc., Canada) and the OpenLab Automator extension of the OpenLab software.

Microscopy

All experiments on the single hexotransporter strains were performed on a Leica DMI 6000B inverted epifluorescense microscope containing a motorized xy stage. Images were taken using a 14-bit dynamic range EM-CCD camera (C9100-12, Hamamatsu Photonics, Japan). For single cell trapping an extension of the 1070-nm ytterbium fiber laser (YLD-5-LP-IPG laser) was used. A fluorescence light source (EL 6000, Leica Microsystems, Germany) was used together with a GFP filter cube (472/30 nm exciter, 520/35 nm emitter, and 495LP dichroic mirror, Semrock IDEX corp., IL) and a mCherry filter cube (560/40 nm exciter, 630/75 nm emitter, and 585LP dichroic mirror, ET-texRed, Chroma, VT). The exposure times used were 27 ms, 150 ms and 300 ms for the transmission, mCherry and GFP states, respectively.

Cell imaging

Seven images with an axial distance of 0.8 µm were acquired in transmission and fluorescent light. The acquisition time of these images at each time point was ±30 sec. Images were acquired at different time points (at 30 sec before the shift, at the shift, at 60 sec, every 60 sec for 420 sec, every 120 sec for 360 sec, and every 180 sec for 360 sec after the shift), adding up to an overall experiment time of 20 min.

Data analysis

The transmission light images were used to calculate the cell size and segmentation. For the segmentation of the whole cell the software Cellstat was used [3]. The fluorescent images from the mCherry filter was the nuclear size and localization obtained, and images from the GFP filter where used to obtain the dynamics of the Mig1 localization. The open source software CellStress was used for both detection of the nucleus, and measurement of GFP fluorescence intensity [4]. Fluorescent intensity data are presented as the ratio of the GFP intensity in the

entire nucleus relative to the intensity in the whole cytosol over time. Images analysis of the experiments on the Hxt7-GFP strain is performed with ImageJ [5].

Model description

The dynamical model consists of 8 species, 12 reactions and 18 parameters and has three main parts, namely the activity of glucose, the activity of Snf1 and the activity of Mig1. In Equation 1, the three scalars $\mathrm{HXT1a}$, $\mathrm{HXT4a}$ and $\mathrm{HXT7a}$ are introduced in order to account for the three data sets, that is the HXT1, the HXT7 and the WT data sets. These three scalars are binary variables and the following three sets of the variables were included in the modelling of the Mig1 localization. The set $(\mathrm{HXT1a} \quad \mathrm{HXT4a} \quad \mathrm{HXT7a}) = (1 \quad 0 \quad 0)$ is used in order to model the dynamics of the HXT1 strain, the set $(\mathrm{HXT1a} \quad \mathrm{HXT4a} \quad \mathrm{HXT7a}) = (0 \quad 0 \quad 1)$ is used in order to model the dynamics of the HXT7 strain and the set $(\mathrm{HXT1a} \quad \mathrm{HXT4a} \quad \mathrm{HXT7a}) = (1 \quad 1 \quad 1)$ is used in order to model the dynamics of the WT strain.

Estimating the fixed effect parameter Θ

We estimated the parameters by searching for the parameters that minimize the distance between the measured and simulated output. In which $\hat{y}(\theta) = \frac{nMig1(\theta)+nMig1p(\theta)}{cMig1(\theta)+cMig1p(\theta)}$ denote the simulated output and y is the measured output, the best suited parameter would solve the minimization problem:

$$\boldsymbol{minimize\ LS(\theta) = ||y - y(\theta)||^2} \qquad \text{Equation 10}$$

where $||\cdot||$ is the Euclidian distance measure. The solution $\bar{\theta}$ to the problem in Equation 10 is called the *least square estimate*[6]. To find the optimal solution vector we applied a descent algorithm belonging to the class of *continuous optimization* technique - a gradient based method that searches for a minima in the search space by "walking" in the direction of the gradient of the least square $\nabla LS(\theta)$ (note that a minima is characterized by the property $\nabla LS(\theta) = 0 \Longrightarrow \theta\ is\ minima$) [7-9]. However, in order to narrow down the search space we added constraints to the parameter vector θ with the form $a \leq \theta \leq b$ where $a, b \in \mathbb{R}_+^{18}$ are two vectors that contain the lower and upper bounds of the parameters contained in θ (Table 4). By adding the constraints to the minimization problem in Equation 10, it can be reformulated as in Equation 11.

$$\begin{aligned} \boldsymbol{minimize\ LS(\theta)} \quad &= \boldsymbol{||y - y(\theta)||^2} \\ \boldsymbol{subject\ to} \quad & \\ &\boldsymbol{\theta - b \leq 0} \\ &\boldsymbol{\theta - a \geq 0} \end{aligned}$$

Equation 11

In Equation 11 the optimality conditions for the constrained problem are called the Karush Khun Tucker (*KKT) conditions* and are conditioned by adding the constraint functions to the objective function using Lagrange multipliers [9].

Table S4. Upper and lower bounds for every parameter. The upper and lower bounds for Vmg1, Km1, Vmg4, Km4, Vmg7 and Km7 are dependent on the external glucose concentration, in this table there are given for the upshift to 220 mM.

Parameter	Upper bound	Lower bound	Units	Source
Kim2	0.1	0.001	$[s^{-1}]$	Estimated
Kex2	0.1	0.01	$[s^{-1}]$	Estimated
Vmsa	10^9	10^8	$\left[\frac{\mu M}{s}\right]$	Estimated
Kim1	0.1	0.01	$[s^{-1}]$	Estimated
Vmg4	1.3958×10^8	1.2010×10^8	$\left[\frac{\mu M}{s}\right]$	[10]
Vmg7	1.4347×10^9	1.2703×10^9	$\left[\frac{\mu M}{s}\right]$	[1]
Vmm	10^9	10^8	$[s^{-1}]$	Estimated
Vmg1	1.0236×10^10	7.2061×10^9		[1]
Kex1	0.1	0.01	$[s^{-1}]$	Estimated
Km7	3 100	1 900	$[\mu M]$	[1]
Ka1	10^5	10^4	$[\mu M]$	Estimated
Ki2	10^5	10^4	$[\mu M]$	Estimated
Km1	324 000	176 000	$[\mu M]$	[1]
Km4	6 500	5 900	$[\mu M]$	[10]
Vmsi	0.0463	0.0313	$[s^{-1}]$	Estimated
Vmd	10^9	10^8	$\left[\frac{\mu M}{s}\right]$	Estimated
Ki1	10^5	10^4	$[\mu M]$	Estimated

Ka2	10^5	10^4	$[\mu M]$	Estimated

Estimating the covariance matrix σ

Furthermore, given the above notation, the covariance matrix is estimated using Equation 12 below [6, 11].

$$\boldsymbol{\sigma} = \boldsymbol{s}^2 \cdot (\boldsymbol{X}^T \cdot \boldsymbol{X})^{-1} \qquad \text{Equation 12}$$

In Equation 12, s^2 is the estimate for the overall variance and is given by $s^2 = \frac{LS(\bar{\theta})}{n-p}$ where n is the number of observations and p is the number of estimated parameters. For a dynamical system, the matrix X (which has the dimensions $X \in \mathbb{R}^n \times \mathbb{R}^p$) in Equation 12 is the Jacobian matrix [11]. To solve the symmetric matrix $(X^T \cdot X)^{-1}$ it has to be estimated based on the stoichiometric matrix of the ODE model.

Construction of the covariance matrix

In order to quantify the dependence between different variables, a covariance matrix is often constructed. This matrix depends on the terms variance and covariance. The variance measures the spread of a data set or a parameter, while the covariance measures the nature of the dependence between two different variables. As can be seen in equation 12, the calculation of the covariance matrix depends on the estimation of the matrix $(X^T \cdot X)^{-1}$ where X is the Jacobian matrix of the system. However, since the matrix X is unknown, the matrix $(X^T \cdot X)^{-1}$ has to be estimated in another manner. Note that the matrix $(X^T \cdot X)^{-1}$ has to be quadratic (in this case it is a 18×18-matrix since the parameter vector θ is a 18×1-vector), it is symmetric (i.e. $((X^T \cdot X)^{-1})^T = (X^T \cdot X)^{-1}$ which means that each row is equivalent to the corresponding column) and it is a positive semidefinite matrix (i.e. $\lambda_i \geq 0 \forall i \in \{1, \dots, 18\}$ where λ_i are all the eigenvalues of the matrix$(X^T \cdot X)^{-1}$).

Given the systems of ODE's that governs the dynamics of the Mig1-Snf1 pathway (Equation1-8) , the relationship between the various species (rows) and the reactions in the system (columns) can be illustrated by constructing the stochiometric matrix (Table 5).

Table S5. The stochiometric matrix. The columns represent the reactions and the rows represent the species. The values in the matrix are either -1, 0 or 1 depending on if the species is consumed (value -1), not present (value 0) or produced in the respective reaction (value 1).

	r_1	r_2	r_3	r_4	r_5	r_6	r_{7a}	r_{7b}	r_8	r_{1i}	r_{4i}	r_{7i}
**cMig1p**	+1	-1	0	0	0	0	0	0	0	0	0	0
**cMig1**	0	+1	-1	0	0	0	0	0	0	0	0	0
$\boldsymbol{G_{in}}$	0	0	0	-1	0	0	0	0	0	+1	+1	+1

**cSnf1**	0	0	0	0	+1	-1	0	0	0	0	0	0
**cSnf1p**	0	0	0	0	-1	+1	-1	+1	0	0	0	0
**nMig1**	0	0	+1	0	0	0	0	0	-1	0	0	0
**nMig1p**	-1	0	0	0	0	0	0	0	+1	0	0	0
**nSnf1p**	0	0	0	0	0	0	+1	-1	0	0	0	0

Now, given the information in the stoichiometric matrix the covariance matrix is constructed by using the following five rules.

1) The covariance between two parameters that correspond to two reactions that are not connected in the stoichiometric matrix are set to zero.
2) All the variances are set to the value “var”.
3) All parameters that are involved in the *same* reaction are set to the value “+cov” (that is *positive covariance*) which means that if one parameter in the reaction increase so does the other.
4) All the parameters that are related by the *opposite* sign in the stoichiometric matrix are assigned a value “+cov” (that is *positive covariance*).
5) All the parameters that are related by the *same* sign in the stoichiometric matrix are assigned a value “-cov” (that is *negative covariance*).

Using the above rules and by listing the parameters (excluding Kd) in the same order as they appear in Table 2 (in the main text), the estimate of the matrix $(X^T \cdot X)^{-1}$ can be constructed (Equation 13). For each row, and by definition for each column, the elements of the matrix $(X^T \cdot X)^{-1}$ can be interpreted as follows. The element $1{\times}1$ is proportional to the interaction between the first and the first parameter, the element $1{\times}2$ is proportional to the interaction between the first and the second parameter, the element $1{\times}3$ is proportional to the interaction between the first and the third parameter etcetera. Subsequently, all diagonal elements will be denoted “var” (as in variance) and all the remaining nonzero elements will be denoted “cov” (as in covariance). Note that a negative covariance corresponds to a negative correlation or inverse proportionality between the two parameters while a positive covariance corresponds to a positive correlation or proportionality between the two parameters. Thus, by knowing how the reactions are connected in the system depicted in Figure 1, the interaction between the various reactions constants can be estimated by connecting each reaction constant with its corresponding reaction through the information stored in Table 5.

Equation 13

$$\left(X^{T} \cdot X\right)^{-1} =$$

$$\begin{pmatrix}
\mathrm{var} & +\mathrm{cov} & +\mathrm{cov} & 0 & 0 & 0 & 0 & 0 & 0 & 0 & 0 & 0 & 0 & 0 & 0 & 0 & +\mathrm{cov} & +\mathrm{cov} \\
+\mathrm{cov} & \mathrm{var} & +\mathrm{cov} & +\mathrm{cov} & 0 & 0 & 0 & 0 & 0 & 0 & 0 & 0 & 0 & 0 & 0 & 0 & 0 & 0 \\
+\mathrm{cov} & +\mathrm{cov} & \mathrm{var} & +\mathrm{cov} & 0 & 0 & 0 & 0 & 0 & 0 & 0 & 0 & 0 & 0 & 0 & 0 & 0 & 0 \\
0 & +\mathrm{cov} & +\mathrm{cov} & \mathrm{var} & 0 & 0 & 0 & 0 & 0 & 0 & 0 & 0 & 0 & 0 & 0 & 0 & +\mathrm{cov} & +\mathrm{cov} \\
0 & 0 & 0 & 0 & \mathrm{var} & +\mathrm{cov} & -\mathrm{cov}^{\star} & -\mathrm{cov}^{\star} & -\mathrm{cov}^{\star} & -\mathrm{cov}^{\star} & 0 & 0 & 0 & 0 & 0 & 0 & 0 & 0 \\
0 & 0 & 0 & 0 & +\mathrm{cov} & \mathrm{var} & -\mathrm{cov}^{\star} & -\mathrm{cov}^{\star} & -\mathrm{cov}^{\star} & -\mathrm{cov}^{\star} & 0 & 0 & 0 & 0 & 0 & 0 & 0 & 0 \\
0 & 0 & 0 & 0 & -\mathrm{cov}^{\star} & -\mathrm{cov}^{\star} & \mathrm{var} & +\mathrm{cov} & -\mathrm{cov}^{\star} & -\mathrm{cov}^{\star} & 0 & 0 & 0 & 0 & 0 & 0 & 0 & 0 \\
0 & 0 & 0 & 0 & -\mathrm{cov}^{\star} & -\mathrm{cov}^{\star} & +\mathrm{cov} & \mathrm{var} & -\mathrm{cov}^{\star} & -\mathrm{cov}^{\star} & 0 & 0 & 0 & 0 & 0 & 0 & 0 & 0 \\
0 & 0 & 0 & 0 & -\mathrm{cov}^{\star} & -\mathrm{cov}^{\star} & -\mathrm{cov}^{\star} & -\mathrm{cov}^{\star} & \mathrm{var} & +\mathrm{cov} & 0 & 0 & 0 & 0 & 0 & 0 & 0 & 0 \\
0 & 0 & 0 & 0 & -\mathrm{cov}^{\star} & -\mathrm{cov}^{\star} & -\mathrm{cov}^{\star} & -\mathrm{cov}^{\star} & +\mathrm{cov} & \mathrm{var} & 0 & 0 & 0 & 0 & 0 & 0 & 0 & 0 \\
0 & 0 & 0 & 0 & 0 & 0 & 0 & 0 & 0 & 0 & \mathrm{var} & +\mathrm{cov} & +\mathrm{cov} & +\mathrm{cov} & -\mathrm{cov} & +\mathrm{cov} & 0 & 0 \\
0 & 0 & 0 & 0 & 0 & 0 & 0 & 0 & 0 & 0 & +\mathrm{cov} & \mathrm{var} & +\mathrm{cov} & +\mathrm{cov} & -\mathrm{cov} & +\mathrm{cov} & 0 & 0 \\
0 & 0 & 0 & 0 & 0 & 0 & 0 & 0 & 0 & 0 & +\mathrm{cov} & +\mathrm{cov} & \mathrm{var} & +\mathrm{cov} & +\mathrm{cov} & -\mathrm{cov} & 0 & 0 \\
0 & 0 & 0 & 0 & 0 & 0 & 0 & 0 & 0 & 0 & +\mathrm{cov} & +\mathrm{cov} & +\mathrm{cov} & \mathrm{var} & +\mathrm{cov} & -\mathrm{cov} & 0 & 0 \\
0 & 0 & 0 & 0 & 0 & 0 & 0 & 0 & 0 & 0 & -\mathrm{cov} & -\mathrm{cov} & +\mathrm{cov} & +\mathrm{cov} & \mathrm{var} & +\mathrm{cov} & 0 & 0 \\
0 & 0 & 0 & 0 & 0 & 0 & 0 & 0 & 0 & 0 & +\mathrm{cov} & +\mathrm{cov} & -\mathrm{cov} & -\mathrm{cov} & +\mathrm{cov} & \mathrm{var} & 0 & 0 \\
+\mathrm{cov} & 0 & 0 & +\mathrm{cov} & 0 & 0 & 0 & 0 & 0 & 0 & 0 & 0 & 0 & 0 & 0 & 0 & \mathrm{var} & 0 \\
+\mathrm{cov} & 0 & 0 & +\mathrm{cov} & 0 & 0 & 0 & 0 & 0 & 0 & 0 & 0 & 0 & 0 & 0 & 0 & 0 & \mathrm{var}
\end{pmatrix}$$

Note that the value of “$cov^{\star}$” in Equation 13 are zero for the HXT1 and HXT7 data sets while it is nonzero in the WT data sets. These covariance elements correspond to the interaction between the various hexose transporters in the model and consequently they depend on the data set.

In order to generate the simulations in Figure 8, the variance is assigned the arbitrary value of 1, that is $var = 1$. Furthermore, it is of great importance to choose the values of the nonzero covariance elements (values of “*cov*” in Equation 13) in relation to the variance elements (values of “var” in Equation 13) that will result with a *positive semidefinite* matrix. This can be achieved by using the Gershgorin's Theorem, [12], which states that the eigenvalues λ_i (where $i \in \{1, \dots, 18\}$) of $(X^T \cdot X)^{-1}$ live in balls centered at the diagonal element “*var*” and with radius equal to the sum of the absolute value of the covariance elements which is written as $\lambda_i \in B(var, \sum_{j=1, j\neq i}^{18} |x_{ij}|)$ where $i \in \{1, \dots, 18\}$ and x_{ij} is the element of $(X^T \cdot X)^{-1}$ that is found in the ith row and the jth column. Thus, in order to have a positive eigenvalue λ_i it is necessary to impose the condition $var \geq \sum_{j=1, j\neq i}^{18} |x_{ij}|$, and by studying the matrix in Equation 13 it is clear that the largest value (for any $i \in \{1, \dots, 18\}$) that the sum $\sum_{j=1, j\neq i}^{18} |x_{ij}|$ can take is $(5 \cdot cov)$. Thus provided that $var = 1$, choosing the covariance to $cov = \frac{1}{5}$ will result in a positive semidefinite matrix.

Parameter perturbation and model variation In order to test the robustness of the model sensitivity analysis and model variation analysis were conducted. Two different values of the glucose degradation constant Kd have been implemented, namely $Kd_1 \sim 10^{-3}$ and $Kd_2 \sim 10^{6}$ in combination with two different initial conditions that differed merely in how the initial Mig1-localization was distributed. The first initial condition, denoted *Initial*$_1$, had zero phosphorylated Mig1, that is cMig1p = nMig1p = 0, while the second initial condition, denoted *Initial*$_2$, had equal amounts of phosphorylated and unphosphorylated Mig1, that is cMig1 = cMig1p and nMig1 = nMig1p.

The results in Table 6 show that the value of the degradation constant Kd and the initial conditions have little effect on the least square in the parameter estimation procedure. This implies that how the initial conditions and the

parameter Kd are chosen is of little relevance regarding the estimation of the remaining parameters, and therefore these should be chosen by considering the biological properties at hand. Since the constant Kd_2 results in the most reasonable steady state intracellular glucose concentration, $G_p(t = \infty) \sim 10^3$ μM and the second initial condition *$Initial_2$* that corresponds to the most biologically relevant initial condition (since the amount of phosphorylated protein is not zero) was implemented subsequently.

The purpose of varying the model is to determine the magnitude of the change in the output of a model in response to a change in a certain parameter of the model. In this particular model, each parameter in the fixed effect parameter vector Θ has been perturbed individually by multiplying the parameter of interest with a scalar of the value exp (s^2).For simplicity we denote the perturbed vector Θ_{per_i} where $i \in \{1, \dots, 18\}$ is the index of the parameter that is being perturbed. Given the above notation, a measure of the change in the output in response to the perturbation in the model is given by Equation 14:

$$e_i = \frac{\left\| \hat{y}(\bar{\Theta}) - \hat{y}(\hat{\Theta}_{per_i}) \right\|}{l} \qquad \text{Equation 14}$$

where e_i is the mean model variation error of parameter $i \in \{1, \dots, 18\}$ and l is the number of time points for which the output has been measured. By comparing the mean model variation error in Equation 14 for each parameter it is possible to determine the parameter in the model that is responsible for the cell-to-cell variability in the measured output.

Table S6. Sensitivity Analysis. The least square values (LS) of two different values of the glucose degradation constant $Kd_1 \sim 10^{-3}$ and $Kd_2 \sim 10^6$ with two different initial conditions are given. The first initial condition, *$Initial_1$*, had zero phosphorylated Mig1, that is cMig1p = nMig1p = 0, while the second initial condition, *$Initial_2$*, had equal amounts of phosphorylated and unphosphorylated Mig1, that is cMig1 = cMig1p and nMig1 = nMig1p.

**Least square value, LS**				
**Gex [mM]**	(Kd_1,$Initial_1$)	(Kd_1,$Initial_2$)	(Kd_2,$Initial_1$)	(Kd_2,$Initial_2$)
**HXT7 data**				
220	3.834417	3.835097	3.834370	3.835050
55	2.937918	2.937862	2.937918	2.937862
27.5	3.898142	3.897893	3.898141	3.897892
11	4.627039	4.626970	4.627039	4.626970

2.75	5.373709	5.373644	5.373709	5.373644
0	0.390279	0.390275	0.390279	0.390275
**HXT1 data**				
220	39.551342	39.551222	39.551341	39.551221
55	33.373048	33.372956	33.373048	33.372956
27.5	1.665347	1.665321	1.665346	1.665321
11	2.661176	2.661053	2.661176	2.661053
2.75	3.655134	3.655117	3.655134	3.655117
0	0.211134	0.211129	0.211134	0.211129
**WT data**				
220	12.116645	12.116561	12.116644	12.116560
55	4.557102	4.557085	4.557102	4.557085
27.5	2.430639	2.430716	2.430584	2.430661
11	0.693698	0.693666	0.693699	0.693666
2.75	1.543187	1.543168	1.543187	1.543168
0	0.148027	0.148072	0.148027	0.148072

References

1. Elbing, K., et al., *Role of hexose transport in control of glycolytic flux in Saccharomyces cerevisiae.* Appl Environ Microbiol, 2004. **70**(9): p. 5323-30.
2. Snowdon, C., C. Hlynialuk, and G. van der Merwe, *Components of the Vid30c are needed for the rapamycin-induced degradation of the high-affinity hexose transporter Hxt7p in Saccharomyces cerevisiae.* FEMS Yeast Res, 2008. **8**(2): p. 204-16.
3. Kvarnstrom, M., et al., *Image analysis algorithms for cell contour recognition in budding yeast.* Opt Express, 2008. **16**(17): p. 12943-57.
4. Smedh, M., et al. *CellStress - open source image analysis program for single-cell analysis*. 2010.
5. Schneider, C.A., W.S. Rasband, and K.W. Eliceiri, *NIH Image to ImageJ: 25 years of image analysis.* Nat Methods, 2012. **9**(7): p. 671-5.
6. Rice, J.A., *Mathematical statistics and data analysis*. Vol. 3. 2007, Belmont, Calif: Thomson Brooks/Cole.
7. Andréasson, N., et al., *An introduction to continuous optimization: foundations and fundamental algorithms*. Vol. 2., [rev.]. 2013, Lund: Studentlitteratur.
8. Lasdon, L.S., *Optimization theory for large systems*. 2002, Mineola, N.Y: Dover Publications.

9. Snyman, J., *Practical Mathematical Optimization*. 1 ed. Applied Optimization. Vol. 97. 2005: Springer US. XX, 258.
10. Maier, A., et al., *Characterisation of glucose transport in Saccharomyces cerevisiae with plasma membrane vesicles (countertransport) and intact cells (initial uptake) with single Hxt1, Hxt2, Hxt3, Hxt4, Hxt6, Hxt7 or Gal2 transporters.* FEMS Yeast Res, 2002. **2**(4): p. 539-50.
11. Seber, G. and C.J. Wild, *Nonlinear Regression*. 1989, New York: John Wiley & Sons.
12. Elsner, L., *Geršgorin and His Circles*. 2006, Mathematical Association of America: Washington. p. 379-381.
