# Supplementary material for: Single-cell study links metabolism with nutrient signaling and reveals sources of variability
Source: BMC Syst Biol. 2017 Jun 5;11:59. doi: 10.1186/s12918-017-0435-z (PMC5460408; doi:10.1186/s12918-017-0435-z)
Supplement: Supplementary file 11 — A collection of all parameters in the model. (PDF 37 kb) [file 12918_2017_435_MOESM11_ESM.pdf]

Parameter	Reaction	Expression for reaction	Description of parameter	Unit	Included in Parameter Estimation (Yes/No)
Kex2	r_1	$Kex2 \cdot nMig1p$	Export of Mig1p out of the nucleus	$[s^{-1}]$	Yes
Vmd	r_2	$\frac{Vmd \cdot cMig1p}{Ka2 + cMig1p}$	Dephosphorylation of cytosolic Mig1	$\left[\frac{\mu M}{s}\right]$	Yes
Ka2	r_2	$\frac{Vmd \cdot cMig1p}{Ka2 + cMig1p}$	Dephosphorylation of cytosolic Mig1	$[\mu M]$	Yes
Kim2	r_3	$Kim2 \cdot cMig1$	Import of Mig1 into the nucleus	$[s^{-1}]$	Yes
Vmg1	r_{1i}	$\frac{Vmg1 \cdot G_{ex}}{Km1 + G_{ex}}$	Import of Glucose using HXT1	$\left[\frac{\mu M}{s}\right]$	Yes<br>(for HXT1 data)
Km1	r_{1i}	$\frac{Vmg1 \cdot G_{ex}}{Km1 + G_{ex}}$	Import of Glucose using HXT1	$[\mu M]$	Yes<br>(for HXT1 data)
Vmg4	r_{4i}	$\frac{Vmg4 \cdot G_{ex}}{Km4 + G_{ex}}$	Import of Glucose using HXT4	$\left[\frac{\mu M}{s}\right]$	Yes<br>(for HXT4 data)
Km4	r_{4i}	$\frac{Vmg4 \cdot G_{ex}}{Km4 + G_{ex}}$	Import of Glucose using HXT4	$[\mu M]$	Yes<br>(for HXT4 data)
Vmg7	r_{7i}	$\frac{Vmg7 \cdot G_{ex}}{Km7 + G_{ex}}$	Import of Glucose using HXT7	$\left[\frac{\mu M}{s}\right]$	Yes<br>(for HXT7 data)
Km7	r_{7i}	$\frac{Vmg7 \cdot G_{ex}}{Km7 + G_{ex}}$	Import of Glucose using HXT7	$[\mu M]$	Yes<br>(for HXT7 data)
Vmsi	r_5	$\frac{Vmsi \cdot cSnf1p \cdot G_{in}}{Ki1 + cSnf1p}$	Dephosphorylation of cytosolic Snf1	$[s^{-1}]$	Yes
Ki1	r_5	$\frac{Vmsi \cdot cSnf1p \cdot G_{in}}{Ki1 + cSnf1p}$	Dephosphorylation of	$[\mu M]$	Yes

			cytosolic Snf1		
Vmsa	r_6	$\frac{Vmsa \cdot cSnf1}{Ka1 + cSnf1}$	Phosphorylation of cytosolic Snf1	$\left[\frac{\mu M}{s}\right]$	Yes
Ka1	r_6	$\frac{Vmsa \cdot cSnf1}{Ka1 + cSnf1}$	Phosphorylation of cytosolic Snf1	$[\mu M]$	Yes
Kim1	r_{7a}	$Kim1 \cdot cSnf1p$	Import of phosphorylated Snf1 into the nucleus	$[s^{-1}]$	Yes
Kex1	r_{7b}	$Kex1 \cdot nSnf1p$	Export of phosphorylated Snf1 out of the nucleus	$[s^{-1}]$	Yes
Vmm	r_8	$\frac{Vmm \cdot nMig1 \cdot nSnf1p}{Ki2 + nMig1}$	Phosphorylation of Mig1 in the nucleus	$[s^{-1}]$	Yes
Ki2	r_8	$\frac{Vmm \cdot nMig1 \cdot nSnf1p}{Ki2 + nMig1}$	Phosphorylation of Mig1 in the nucleus	$[\mu M]$	Yes
Kd	r_4	$K_d \cdot G_{in}$	Degradation G_{in} through metabolism	$[s^{-1}]$	No

Table S3. A collection of all parameters in the model.

The parameters are shown in column 1, the reaction in which the parameter occurs is shown in column 2, the expression for the reaction is shown in column 3, the meaning of the parameter is shown in column 4, the unit of the parameter is shown in column 5 and whether or not the parameter is included in the parameter estimation is shown in column 6.
